# Important challenges for coordination and inter-municipal cooperation in health care services: a Delphi study

**DOI:** 10.1186/1472-6963-13-451

**Published:** 2013-10-30

**Authors:** Elisabeth Holen-Rabbersvik, Tom Roar Eikebrokk, Rune Werner Fensli, Elin Thygesen, Åshild Slettebø

**Affiliations:** 1 Department of Health and Nursing Science, University of Agder, Grimstad, Norway; 2 Department of Information Systems, University of Agder, Kristiansand, Norway; 3 Department of ICT, University of Agder, Grimstad, Norway; 4 Department of Health and Nursing Science, University of Agder, Kristiansand, Norway

**Keywords:** IMC, Delphi, Coordination, Cooperation, Municipality, Health, Reform

## Abstract

**Background:**

Demographical changes have stimulated a coordination reform in the Norwegian health care sector, creating new working practices and extending coordination within and between primary and hospital care, increasing the need for inter-municipal cooperation (IMC). This study aimed to identify challenges to coordination and IMC in the Norwegian health care sector as a basis for further theorizing and managerial advice in this growing area of research and practice.

**Methods:**

A Delphi study of consensus development was used. Experts in coordination and IMC in health care services were selected by the healthcare manager or the councillor in their respective municipalities. In the first round, an expert panel received open-ended questions addressing possible challenges, and their answers were categorized and consolidated as the basis for further validation in the second round. The expert panel members were then asked to point out important statements in the third round, before the most important statements ranked by a majority of the members were rated again in the fourth round, including the option to explain the ratings. The same procedure was used in round five, with the exception that the expert panel members could view the consolidated results of their previous rankings as the basis for a new and final rating. The statements reaching consensus in round five were abstracted and themed.

**Results:**

Nineteen experts consented to participate. Nine experts (47%) completed all of the five rounds. Eight statements concerning coordination reached consensus, resulting in four themes covering these challenges: different culture, uneven balance of power, lack of the possibility to communicate electronically, and demanding tasks in relation to resources. Three statements regarding challenges to IMC reached consensus, resulting in following themes: coopetition, complex leadership, and resistance to change.

**Conclusions:**

This study identified several important challenges for coordination and it supports previous research. IMC in health care services deals with challenges other than coordination, and these must be addressed specifically. Our study contributes to extended knowledge of theoretical and practical implications in the field of coordination and IMC in health care sector.

## Background

In European countries there will be substantial demographic changes resulting in challenges of costs and capacity in the health care services. To meet these challenges many European countries have implemented new working practices.

The “Coordination reform” was implemented in the Norwegian health care services in 2012. This reform entails that more advanced treatment shall occur in the municipalities through new working practices, such as extended coordination within primary care and between primary and hospital care. In this study, the health care sector is considered as hospital services organized at the regional level and primary health care services organized in the municipalities, and both are publicly funded. In Norway, there are 429 municipalities, and more than half of these have less than 5000 inhabitants. For municipalities to manage both the specialized and increased amount of tasks, the coordination reform entails an increased need for inter-municipal cooperation (IMC) between small municipalities. It is expected that the municipalities will implement IMC when necessary.

In the field of collaboration/coordination, research has targeted the need for inter-organizational integration in health care services, and has pointed to the fragile and volatile nature of inter-sectorial collaboration [1]. Management, as well as commitment and support from actors, are mentioned as essential when delivering integrated care. Other important challenges for integrated care are the institutional context, such as the financing system and legislation [2]. Moreover, organizational culture is found to be of great importance to inter-sectorial collaboration [3] and important barriers are identified in professional tribalism, and status and power differentials [4]. In a previous report, 10 challenges were identified that must be addressed to achieve successful collaboration, including careful preparation and organization by the leaders, and supportive home management [5].

Research based on neo-institutional theory has shown that obstacles in tackling health care reforms have less to do with “what to do” than “how to do it” [6]. Other research has shown that health reform complexity is often noticed by the planners, but are not taken as seriously as they should be [7]. This lack of attention towards complex problems in the health reform is also addressed in a study by Glouberman and Zimmerman [8].

Many reports on IMC are non-scientific and in general, there is a scarcity of systematic research targeting IMC. In “Inter-Municipal Cooperation in Europe” [9], the authors conclude that research into the performance of IMC and into factors determining success and failure should be at the top of future research agendas. In 2006, a report [10] showed that inter-municipal work in Norway is lacking political management, control, and overview, concluding that the situation could undermine local democratic control of basic public services, and measures should be implemented to enhance the visibility, knowledge, and awareness of IMC.

A study by Haveri [11] found that even though the need for cooperation is acknowledged in most surveys, practical steps are difficult to carry out because of complexity in inter-organizational action.

Given the establishment of new collaboratives with substantial differences in interests, Øvretveit et al. [5] suggest that more research is required on this issue.

To address this gap in the literature, the purpose of this study is to identify the most important challenges for coordination and IMC in the health care services, from the viewpoint of experts in the municipalities.

## Methods

A Delphi study among Norwegian experts was conducted to identify and prioritize challenges that might hinder coordination and IMC in health care services in Norway. The study was conducted from May to August, 2012. In the present study, IMC includes cooperative arrangements between municipalities and not between municipalities and other organizations. The phrase “coordination” is used, referring to the “coordination reform”. The meaning of coordination in this context is explained in a report to the Storting (Norwegian Parliament) [12] where the term “coordination” is used to describe the ability of different healthcare services to unite tasks to reach a common goal, and to complete the tasks in a coordinated and rational way. The phrase “cooperation” is used in the context of IMC, and is an established phrase in the literature [13,14].

According to Powell [15], the Delphi technique has the advantage of being a democratic and structured approach, which exploits the collective knowledge of the participants. The Delphi technique is thus a promising method for facilitating communication and seeking consensus within different groups. In essence, the procedure comprises several sequences of questionnaires or rounds to a group of experts within a specific field, with controlled feedback from the researcher. The Delphi technique attempts to seek the most reliable consensus of opinion for a group of experts [16,17]. This Delphi study was conducted in three steps; it started with open-ended questions, qualitative data were validated, and it finished with three rounds of a consensus process.

As a first step, a pilot study was conducted to test how SurveyXact could support the Delphi method, test wording and response format, and finally, how much time was required in each round. Five informants were included, four with relevant experience of IMC, and one with research experience related to IMC. The pilot study was conducted in four rounds (response rate round 1, 5/5; round two, 5/5; round three, 3/5; and round four, 3/5.)

The pilot study revealed uncertainty in the interpretation of terms, such as “Delphi” and “panel group”, and resulted in some changes in formulations, questioning and lay out, as well as improvements in the use of open-ended questions, response format, timing of the interviews as well as subsequent survey design.

### Expert panel

A total of 79 municipalities were contacted. Based on data from Statistics Norway, 35 of the contacted municipalities were categorized as large (>20,000 inhabitants) and 43 as small (<5000 inhabitants), as the intentional plan was to compare the two groups. To ensure a variety in the group, the municipalities were evenly distributed with respect to socio-economic criteria as restricted costs per capita and free disposable income per capita. Free disposable income per capita reflects the amount of revenue municipalities have at their disposal after the restricted costs are covered, and indicates the municipalities’ economic freedom.

**Figure 1:**
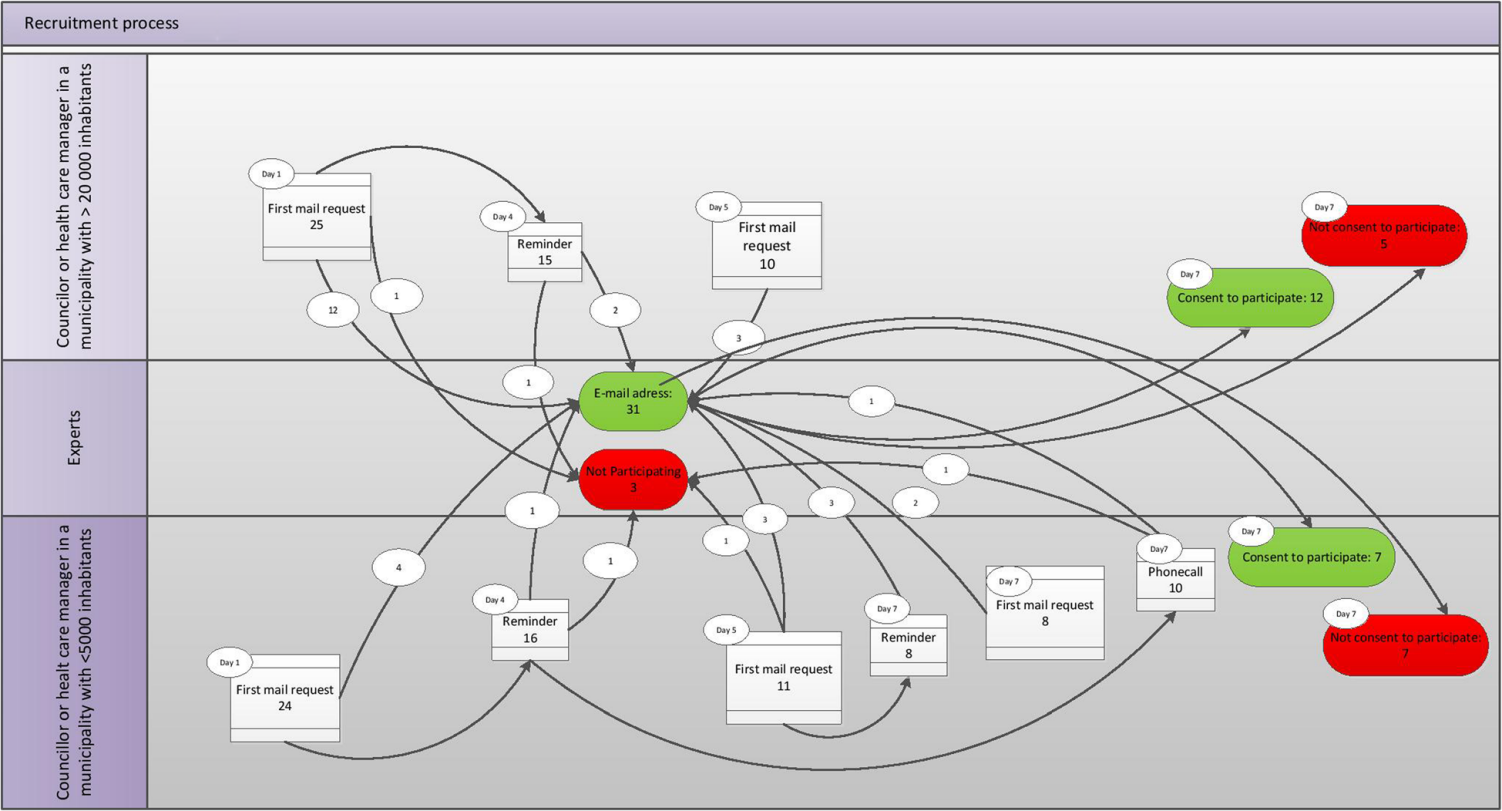
Recruitment process.

Depending on the local municipal structure, health care managers or councillors in the 79 municipalities were contacted by e-mail. We assumed that they had the best knowledge to identify experts of cooperation and IMC in their respective municipalities. The health care managers or councillors were asked to identify and send contact information of the employee in their municipality with the highest competence related to collaboration and IMC in health care services. They were told that the identified person could be a project manager in the field, or a person with good insight by being involved in an implementation and continuation phase of IMC. In some cases, the health care manager or councillor who received the first e-mail could also be this expert. A detailed procedure for recruiting respondents is shown in Figure 1.

Figure 1 shows the number of municipalities that were contacted to identify experts in their respective municipalities. The figure shows the distribution of small and large municipalities, and the number of municipalities that was contacted. As the figure illustrates, a larger amount of small municipalities had to be contacted in several rounds. After seven days with several rounds of recruitment, we had contact information to 31 experts. They received information about the project and link to the first questionnaire. Table 1 shows that a total of 19 experts consented to participate. Out of those, 7 experts were from small municipalities and 12 were from large municipalities. The experts represented municipalities with inhabitants varying from 1500 to 115,000. Table 2 shows the experts position and size of municipality.

**Table 1:** Sample

	**n**	**%**
**Sample**	31	100
**Consent to participate**	19	61,3

**Table 2:** Experts positions

**Size of Municipality**	**Position**
Small	Operating manager, preventive services
Small	Unit manager, nursing and care services
large	Special advisor, faculty of preventive services
large	Municipal Chief Physician
large	Advisor, faculty/support unit
large	Coordinator, the coordination reform
large	Advisor, health care services
large	Coordinator, the coordination reform
large	Project manager, the coordination reform
small	Local authority Executive
small	Coordination contact, Unit Manager, health care.
small	Municipal Chief Physician
large	Local Authority Executive
large	Project Manager, inter-municipal medical center
small	Local Authority Executive
small	Local Project Manager, the coordination reform
large	Unit Manager, health care services
large	Advisor, electronic messaging in care coordination
large	Special Advisor

### Procedure

This study was conducted as a five-round study. SurveyXact was used to distribute questions and collect data. A link to the questionnaire was sent by e-mail to respondents, and the response period was set to 4 working days with a reminder after 2 working days. In a few cases, some respondents stated the need for extended time to answer, and this was accepted. The analysis was conducted in 2–6 working days, and then returned to the experts. Because of the summer vacation, it took almost 2 months between the fourth and fifth rounds. Participants who did not answer a round were excluded from the rest of the study. Table 3 shows the panel participation in the rounds. The language used in this study was Norwegian. One researcher translated the answers to English. The translation was validated by two co-researchers.

**Table 3:** Participation

	**Q-1**	**Q-2**
**Round one (%)**	19 (100)	19 (100)
**Round two (%)**	11 (58)	11 (58)
**Round three (%)**	11 (58)	10 (53)
**Round four (%)**	10 (53)	10 (53)
**Round five (%)**	9 (47)	9 (47)

### Analysis

#### Round 1

In the first round, the experts answered the following open-ended questions:

Q-1. *What challenges do you experience related to coordination with various actors in the health/care services in your community and specialist health care? You should mention point-wise all the challenges you can think of.*

Q-2. *What challenges exist for inter-municipal work in the health care sector? You should mention point-wise all the challenges you can think of.*

In the beginning of the questionnaire, the respondents were given the report to the Storting’s (Norwegian Parliament) description of the term “coordination” [12] to ensure they had a common understanding of the term. Qualitative data were first consolidated, meaning that answers with equal meaning were merged. The data were then analysed to distinguish actual challenges from “consequences” and “measures/success factors”. The answers were also merged based on the overarching theme. This was carried out to validate the researcher’s and expert panels’ understanding of the different answers. Some statements were reformulated to more readily identify the challenge, such as the original statement: *“What activities/tasks we can handle ourselves and what needs cooperation”* was reformulated to *“Lack of clarity related to how to handle ourselves and what needs cooperation”.*

#### Round 2

The expert group in the second round was asked the following questions related to Q-1 and Q-2:

1. *Verify that your answers are sorted under the correct category, to ensure that the meaning of the answer is understood.*

2. *Verify consolidation by ensuring that your opinion will appear under one of the statements (but not necessarily verbatim).*

3. *If you now come up with new challenges, you can add them. You should also consider whether your answers (or the consolidated responses) can be specified more, or incorporated into existing answers. Ask yourself why this is a challenge. For example, “Reform is not fully funded for municipalities.” Why is it a challenge? Do you have no money to hire the necessary expertise? Do patients get lower quality for services? Or are there other reasons that make this a challenge?*

Based on the expert’s responses, minor changes were made, such as adding some new statements, and some statements were reformulated to better communicate the meaning.

#### Round 3

In the third round, the experts were instructed to point out a specified number of the most important statements related to each question. In all of the tasks, the a priori consensus criterion was set at variables selected by more than half of the experts. To avoid context effects, the variables for each theme were distributed in random order. The questionnaire was made in three versions with the themes unequally distributed.

Q-1. *Choose at least 20 variables you think are important challenges related to coordination in health care services. Your answers should be based on the competence you have in your position. The challenges do not have to relate to your own experiences in your municipality.*

Q-2. *Choose at least 10 variables you think are important challenges related to inter-municipal work in health care services. Your answers should be based on the competence you have in your position. The challenges do not have to relate to your own experiences in your municipality.*

In the task related to Q-1, 73 statements identified in round two were presented. One statement identified in round 2 was missing in round three. This statement was presented in round four. A total of 16 statements reached consensus. To avoid ambiguities, the statements were specified by two researchers, ending up with 20 statements. An additional file shows the 74 statements, including the missing statement. Number of times chosen and statements reaching consensus are presented [see Additional file 1].

In the task related to Q-2, 26 statements identified in round two were presented. A total of 13 statements reached consensus. To avoid ambiguity, the statements were specified by two researchers, ending up with 17 statements. One expert identified nine statements. As it was likely that this was a mistake made by the expert, the answers were included in the analysis. It was considered that removing the nine chosen answers had more potential to bias the results, than the lack of one chosen statement. One expert identified three statements, which was regarded as too few to be included in the analysis. This expert answered correctly regarding Q-1, and was therefore invited to the subsequent rounds.

An additional file shows the 26 statements. Number of times chosen and statements reaching consensus are presented [see Additional file 2].

#### Round 4

In round four, the experts were instructed to rate the important variables related to each question on a five point Likert-type scale. To avoid context effects, the variables for each theme were distributed in random order. In addition, they could comment on their explanations for their ratings. The missing statement from round three was also presented, and the experts were instructed to rate this provided they would have chosen it in round three. An additional file shows the statements presented in round four [see Additional file 3].

On a scale from 0–4, where 0 = no extent and 4 = a very large extent, the experts were asked the following questions:

Q-1. *To what extent do you believe that these challenges can hinder coordination in health care?*

Q-2. *To what extent do you think that these challenges can hinder inter-municipal cooperation in health care?*

#### Round 5

In round five, the experts were presented the results from round four. The variables for each theme were distributed in random order. The results were presented as sample maximum and minimum, mode, median, and the consensus value was set as 80% response within two adjacent values. The two adjacent values that had most rates were set as the consensus value. When the two adjacent values were equally distributed, their mean value was set as the consensus value. In addition, the experts were presented with the other experts’ explanations for rating, before they were instructed to re-rate the variables. This step followed the same procedure as in round four.

Agreement among experts in rounds 4 and 5 was analysed using Fleiss kappa statistics, calculated by IBM SPSS statistics 19.

Because the aim in this study was to identify the most important challenges, consensus in round five was set with statements rated as 3 or 4 (where 0 = no extent and 4 = a very large extent) by more than 75% of the experts. Statements reaching this value were abstracted and divided into themes by the researchers.

### Ethics

This study was approved by the Norwegian Social Science Data Services (NSD) (30209) and exempted from ethical approval from a Regional Ethical Committee according to Norwegian law. The experts gave voluntary informed consent to participate.

## Results

In round one and round two, the aim was for experts to identify and validate statements concerning coordination and IMC in health care services. A total of 74 challenges were identified concerning coordination and 26 barriers were identified concerning IMC.

In rounds three, four, and five, the aim was to obtain consensus on the most important challenges concerning coordination in general and IMC in particular in health care services. Eight challenges of coordination obtained consensus. The researchers abstracted the challenges into four themes. Three challenges of IMC obtained consensus. The researchers abstracted the challenges into three themes.

### Methodological result

Fleiss’ kappa was used to measure agreement among the experts. Table 4 shows that value of kappa increased concerning Q-1 and Q-2, indicating that that the principle of a successive broader agreement between experts during the rounds was fulfilled. In round five, the value of kappa in Q-1 was higher than that in Q-2. The expert panel is homogenous because they are all defined as experts in coordination and IMC in their municipality. However, they are heterogeneous with regard to the size of the experts’ municipalities. Obtaining consensus in a heterogeneous expert panel is more difficult. This result suggests that large and small municipalities experience many of the same challenges concerning coordination, but for IMC, their view on what is important varies to a greater extent. The size of the experts’ municipality might be an influencing factor.

**Table 4:** Fleiss Kappa statistics

	**Round four**	**Round five**
**Q 1**	Moderate agreement (kappa = 0,54)	Substantial agreement (kappa = 0,68)
**Q 2**	Fair agreement	Fair agreement
	(kappa = 0,34)	(kappa = 0,38)

### Coordination

In Q-1, eight statements reached consensus. Table 5 shows statistics of statements reaching consensus. These statements were abstracted into overarching themes by the researchers as shown in Table 6.

**Table 5:** Descriptive statistics for statements reaching consensus in round five, Q-1

**Statement rated 3 or 4 by >75%**	**Measure**	**Value**
Different culture between municipal and specialist health care services.	Sample maximum and minimum	2-4
	Median	3
	Mode	3
Specialist health service has focus on diagnosis and treatment, but the municipality has focused on coping and quality of life.	Sample maximum and minimum	2-4
	Median	3
	Mode	3
Lack of electronic communication.	Sample maximum and minimum	2-4
	Median	4
	Mode	4
Lack of common tools for electronic communication.	Sample maximum and minimum	2-4
	Median	4
	Mode	4
The hospital sets the conditions for the process concerning discharge of patients.	Sample maximum and minimum	2-4
	Median	4
	Mode	4
The reform is not fully funded for the municipalities.	Sample maximum and minimum	2-4
	Median	4
	Mode	4
Scarce resources in terms of time.	Sample maximum and minimum	2-4
	Median	3
	Mode	3
Different patient perspective between municipal and specialist health care services.	Sample maximum and minimum	2-4
	Median	3
	Mode	3
Patients discharged are in worse health condition than before.	Sample maximum and minimum	1-4
	Median	3
	Mode	3

**Table 6:** Results Q-1

**Theme**	**Abstraction**	**Statement**
Different cultures	Challenges concerning the experience of different cultures and relationship between municipal and specialist health care services.	Different cultures in municipal and specialist health care services.
		Specialist health service has focus on diagnosis and treatment, but the municipality has focused on coping and quality of life.
		Different patient perspective in municipal and specialist health care services.
Uneven balance of power	Challenges concerning experience of being the inferior part.	The hospital sets the conditions for the process concerning discharge of patients.
Lack of the possibility to communicate electronically	Challenges concerning lack of possibilities for electronic communication.	Lack of electronic communication.
		Lack of common tools for electronic communication.
Demanding tasks in relation to resources	Challenges concerning the experience of lacking resources and demanding tasks.	Scarce resources in terms of time.
		Patients discharged are in worse health condition than before.

Consensus was reached on three challenges concerning different cultures. Differences were specified based on how to treat the patient and the different views on the patient’s “role” within the hospital and municipal setting. Experience of the hospital taking the lead in a central part of the coordination; the discharge of the patients was also rated as an important challenge.

Consensus was reached on two challenges concerning the lack of possibility to communicate electronically. Lack of common tools for electronic communication and lack of electronic communication were identified challenges in the expert panel. In Norway, juridical restrictions prevent sharing of sensitive information between organizations. Therefore, sensitive information is exchanged as semi-electronic solutions by the use of a secured message exchange with predefined content between health care organizations. The electronic health records (EHR) are characterized by several individual and different solutions, which cannot be integrated. Reasons for the lack of possibility to communicate may vary, but this challenge indicates that this is an important area to be aware of to obtain coordination.

Consensus was also reached on two challenges of the mismatch between available resources and the tasks given. An important challenge was stated that patients who are discharged are in a worse health condition than earlier and that time resources for health professionals in the municipalities are scarce. Coordination reform implies that more demanding tasks will be managed in municipalities and the mismatch between available resources and the tasks given reflect that coordination is not easy to obtain, without having the required resources.

### IMC

In Q-2, three statements reached consensus. The statistics of the statements are presented in Table 7. These statements were abstracted into overarching themes by the researchers and are presented in Table 8.

**Table 7:** Descriptive statistics for statements reaching consensus in round five, Q-2

**Statement rated 3 or 4 by >75%**	**Measure**	**Value**
It could become prestigious to localize the project in own district and closer to the clients. As a result, the choice of municipality to localize services is subject to political debate.	Sample maximum and minimum	2-4
	Median	3
	Mode	3
Political leadership and management of inter-municipal work are demanding (require more than only organizing the municipal services).	Sample maximum and minimum	2-4
	Median	3
	Mode	3 and 4
It is challenging to establish inter-municipal cooperation as it is often more tempting to solve problems alone since this is more flexible, it creates synergy, and expertise that can be applied across the municipality.	Sample maximum and minimum	3-4
	Median	3
	Mode	3

**Table 8:** Results Q-2

**Theme**	**Abstraction**	**Statement**
Coopetition	Challenges concerning wanting to cooperate, but in the same time acting as competitors.	It could become prestigious to localize the project in own district and closer to the clients. As a result, the choice of municipality to localize services is subject to political debate.
Complex leadership	Challenges concerning more demanding tasks on a political level.	Political leadership and management of inter-municipal work are demanding (require more than only organizing the municipal services).
Resistance to change	Challenges concerning resistance to change, “what we have works fine”.	It is challenging to establish inter-municipal cooperation as it is often more tempting to solve problems alone since this is more flexible, it creates synergy, and expertise that can be applied across the municipality.

Consensus was reached on a challenge concerning the competitiveness in IMC. The challenge was based on the statement which pointed out the prestige in delivering service in one’s own district and closer to clients. The paradox of wanting to cooperate and at the same time be a competitor was abstracted into the theme “coopetition”. This challenge refers to a point in the planning phase of IMC, and might hinder establishment of IMC if it is not solved.

Consensus was also reached on a challenge concerning the complex leadership of IMC. The challenge was based on the statement which pointed out the demanding challenges of political leadership and management of IMC in contrast to regular municipal services.

Resistance to change was another identified challenge reaching consensus. The statement leading to this challenge was the conveyed contentment with the present organization and the advantageous ways of solving problems in one’s own municipality. This challenge implies that the effort in establishing IMC is not proportional to expected outcome, compared with what can be managed in one’s own municipality. This makes implementation of IMC challenging, and can hinder establishment of IMC.

## Discussion

### Theoretical implications

The results indicate that challenges in regards to what can hinder IMC are different from the challenges to what can hinder coordination in general in health care services. As a result, there is a need for more research in the field of IMC, and that theories on coordination not necessarily can be transferred to the field of IMC. Theory of coopetition seems particularly relevant as a perspective for understanding challenges of IMC. The municipalities want to cooperate for a service, but they compete on where to place the service because they want it closer to their clients. This cooperative competition is further stimulated by demographic changes during the last 30 years, where 100 Norwegian rural municipalities have experienced a 20% population decrease as a result of migration to urban municipalities [18]. Inter-municipal services located in own municipality might increase the number of jobs, as well as the closeness to the service, thus attracting potential new inhabitants. For municipalities experiencing growth or decline in population, a potential increase of inhabitants is positive. This can explain the competition on where to place the service. The simultaneous presence of cooperation and competition is termed “coopetition” [19]. The concept of coopetition has generally been used in the context of business and game theory [20] but has gained popularity among public policymakers across Europe, the US, and Asia [21,22], probably as a result of changes in the public sector following increased globalization and international agreements. Public governance is characterized by the use of quality indicators and performance management that enable local competition and benchmarking [23], thus explaining the relevance of coopetition theory in the public sector. According to Brandenburger “*Business is cooperating when it comes to creating a pie, and competing when it comes to dividing it up … learning to be comfortable with this duality is key to success*” [20]. This statement is highly relevant and expresses the core in the identified challenge; inter-municipal collaboration increases the value of joint service production but creates challenges in the division of costs and benefits. In this study an identified challenge that potentially can hinder IMC is the satisfaction with how things can be arranged within the present organization. This suggests that the organization of IMC is considered as too cumbersome in relation to the expected gain, or it could have elements of procrastination or denial of future demographical changes. Solving problems based on scientific evidence might help tackle the system, but it could trigger key stakeholders who don’t have acceptability to the solutions. Stakeholders defend what they believe is the proper way of delivering health care, and their opposition shall not be understood only as rational rent seeking [6]. This challenge can be of great importance when central interest groups represent these norms and challenges. According to Contandriopoulos & Brouselle [6], this challenge can best be met using political and governance questions, but not with programmatic questions. This underpins the importance of political management in IMC. Contandriopoulos & Brouselle [4] use neo-institutional theory when concluding that to help understanding the process of health care reform policies a promising avenue is *“… influencing shared norms and values about the nature of health and healthcare in ways that render them compatible with what have been shown to be efficient healthcare delivery models*”. In the context of IMC in health care services, there might not always be an efficient health care delivery model that is transferable to the given context. Because of the unpredictable future, as well as the inherent problems experienced with IMC, we suggest that, in the context of IMC, there should be shared understanding on what is *likely* to be an efficient health care delivery model, not what has been shown to be an efficient health care delivery model. The choice of the model should be based on a broader set of scientific evidence involving not only what currently works, but also future projections, and the municipality’s local knowledge. Based on the result of our study concerning IMC, we suggest that the understanding of the norms and values of health and healthcare should be complemented by a common understanding of future demographic changes in this process of health care reform policies. The special context that is created when demographic changes occur, must be accounted for in developing and choosing the most suited inter municipal health care delivery model. Otherwise, the true challenges of the context (e.g. demographic changes) might be lost and efforts less relevant as norms and models are not compatible. Our study suggests that it is important that political management and key stakeholders with local knowledge are involved in the development of shared understanding, and the theory of coopetition should not be underestimated.

### Practical implications

Our findings have several practical implications. In the coordination reform it is anticipated equality between municipalities and hospitals, but many of the statements dealt with the issue of different perspectives of the services regarding the patient. Traditionally, hospitals have focused on medical recovery, for both its organization and its function. Primary care has largely focused on the patient’s coping and functional level [12]. Primary care is managed by the municipalities, with a total of 429 in Norway. Specialist health care is managed by a total of four Regional Health Organizations. This implies that primary care might experience inferior collaboration with hospitals. The name *specialist* health care might also reflect an authoritarian power. Uneven balance of power is a challenge supported by a study of nurse training in collaborative practise, where the major barriers identified were professional tribalism along with status and power differences [4]. In the Discourse Theory of Habermas, four presuppositions of dialectic procedures are presented [24]. One of them assumes equal voices of participants. Concerning the identified challenge, the voice between the hospital and the municipal staff is not equal. Therefore, the argument in the case of discharging patients does not regard the actual execution of dialectical procedures. As our results show several statements that can hinder coordination regarding inequality between municipalities and hospitals, this must be taken seriously by the policymakers. In coordination reform, the term “coordination” is described as an expression for the health care services’ ability to unite tasks to reach a common goal, as well as the ability to complete the tasks in a coordinated and rational way [12]. This implies that in addition to the services’ traditional goals, they also have to work to reach an overriding common objective with the patient as the central actor. Gray’s view of collaborations is stated as: *“… a process through which parties who see different aspects of a problem can constructively explore their differences and search for solutions that go beyond their own limited vision of what is possible*” [25], and this should be the goal for stakeholders both in hospitals and in the municipalities.

Another challenge that has the potential to hinder coordination is the lack of possibility to communicate electronically. Norway has made an substantial effort to implement ICT in health care services, and is now a leading country in the implementation of the Electronic Health Record (EHR) [26]. However, ICT systems are characterized by several individual and different solutions, which cannot support the need of exchange of health information during patient pathways [26]. At the start of the coordination reform, a report stated that electronic messages are used to a great extent between GPs and primary care. Only a few municipalities use electronic messages in their communication with hospitals. When two statements refer to the lack of electronic communication as a potential barrier to coordination, it can address the lack of electronic tools to communicate, but it might also indicate that electronic messages do not fulfil the need as coordination tools. Electronic communication is an important measure to reach the vision of the coordination reform with “proper treatment – at the right place and right time” [12]. For managers it is important to ensure that electronic communication fulfil the users need in the process of coordination.

Another challenge that can prevent coordination is that tasks are too demanding on resources in the municipalities. The coordination reform implies that more demanding tasks will be managed by the municipalities. The statements from our study conveyed that the patients are in a worse condition when being discharged from hospitals than earlier, and that the health professionals in the municipalities do not have enough time to do their tasks. This might have severe implications for the coordination. The municipalities can choose not to admit the patient, but because of the coordination reform, they must cover costs in the hospital. This can have huge economic consequences for the municipalities. Another solution is for the municipalities to receive the patient even though the capacity is not present, creating a serious threat to patient safety.

The results show that the challenges that can hinder IMC are different from challenges that can hinder coordination in general. A main issue is the complexity of IMC, and this has to be dealt with in other ways than coordination in general. With regard to IMC, scientific knowledge can only be helpful up to a certain point. The complexity of IMC requires political leadership, and cannot only be managed by expert knowledge [11].

Complex problems can also be called “wicked” problems because their potential solutions cannot be transferred and used for similar problems outside of their context? [8]. One study showed that wicked problems during a health reform were observed, but were not taken as seriously as they should have been [7]. In this previous study, complexity and wickedness in the reform were observed, but they were solved as solutions for tame problems [7]. This indicates that even though complexity is identified in the organization, it must be acknowledged and coped with in an appropriate manner.

In the present study’s identified challenges, political level was specifically mentioned. Even though local self-government might profit from IMC compared with centralization, democracy in IMC could be scarce [27]. Decision-making in inter municipal services must be made jointly by cooperative municipalities, and the political involvement in these processes must be recognized. The type of organizational form of the IMC will also have an effect on the level of democracy.

The results of our study show that a challenge was the complex leadership of IMC. Political leadership and management of inter municipal work are demanding, and requires more than only organizing the municipal services. Another important barrier we found was the challenge of establishing IMC, and that is was more tempting to solve things alone. To practitioners it is important to know when it is beneficial to collaborate. The coordination reform says that IMC shall be implemented when necessary, but the results show that it might not always be clear when necessity has occurred. The challenges regarding political management and the benefits of solving things in own municipality must be weighed against the potential benefits of IMC. Research does not provide a satisfying answer to when cooperation can improve implementation, but research has shown that part of the answer is that the impact of cooperation will increases with task complexity. This might indicate that the municipalities shall not cooperate on simple cases that they can manage themselves and get benefits from, but rather save the cooperation to the more complex cases. This might indicate that policy makers in health care services should not always stress the importance of coordination, but rather focus on when it has the potential to provide more benefits than challenges. Due to future demographical changes, we know that health care services cannot be solved as they are today. In the Norwegian context the coordination reform is entailed to meet some of the future demographical changes by giving the municipalities’ greater responsibility for health care services. When it is necessary to work in different ways because of demographical changes, IMC has some clear advantages over other possible solutions, such as territorial reorganization and centralization of local tasks to upper level government. An inter-municipal organization will more easily than other solutions, adapt to new circumstances and developments, and is more capable of dealing with a rapidly changing environment [27]. In order to meet the demographic changes in the future, it might be a good strategy to implement IMC before any complex issues arise with a so called strategic proactive change [28]. The challenge in doing it this way might be that in lack of immediate positive results, the problems of the demanding leadership as well as the lack of benefit when solving problems in once own municipality might be prominent. This demands decision makers to outweigh resisting forces and they have to advocate change strongly to overcome resistance, as recommended in Lewin’s three-step model for change [29].

The results in our study show that an important challenge is where to localize the inter-municipal project, and this is subject to political debate. This challenge is part of the initiation phase of the project and indicates that the need for IMC is agreed upon, but the challenge of location has the potential to hinder the IMC.

The political level is included in two of the three identified challenges of IMC. This is an important finding that clearly differs from the results concerning coordination. This finding indicates that stakeholders at the political level must be emphasized considering IMC.

### Limitations

Because of the low number of respondents, there is a need for more research to both confirm and generalize our findings.

We chose to supplement standard procedures of the Delphi method with the use of a third person to identify the person with the highest competence in coordination and IMC in health care services. This gives the researcher less impact in predefining the qualifications of the expert panel. However, we believe that this technique added to the validity of our data by providing us with participants with the highest level of competence as seen from the context itself rather than from an external researcher. The dropout rate between rounds 1 and 2 was 42%. Panel attrition was expected because the study was voluntary and uncontrollable. In the second round, an extensive amount of data was sent back to the expert panel. Presenting the data clearly was a challenge because of technical restraints in the survey tool. And it was reported by a panel member that it was difficult to get an adequate overview of the categories. This may explain the dropout between the rounds. In Delphi studies aiming to achieve consensus, dropout can be a substantial problem, because one round is largely constructed upon the answers in the previous round. In this study, the aims in rounds one and two were to identify and verify statements, while the aim in rounds 3–5 was consensus achievement. The risk may be that some statements were not properly validated by the experts, and may have had other meanings than conceived by the expert panel or the researcher. However, the consensus process was not subject to a large dropout. Our study does not divide the different organizational forms of IMC, but identifies challenges that encompass all organizational forms of IMC.

## Conclusions

In the current study, eight statements reached consensus as important challenges to coordination in health care services, and three statements reached consensus as important challenges to IMC in health care services. They were abstracted into four and three themes, respectively.

Regarding coordination in health care services, our findings support previous research. The challenges of a different culture, uneven balance of power, lack of possibility to communicate electronically, and a demanding task in relation to available resources are already known from previous research. In the field of IMC, there has been little research in general, especially in the context of health care services. Therefore, our study provides new insight in the field of IMC. The expert groups emphasized different challenges to coordination versus IMC in the health care sector. This indicates that even though both coordination and IMC deal with inter-organizational work, different approaches should be taken to avoid the different challenges. Our main findings are that scientific knowledge only can give scarce information on how to concretely address challenges of IMC. Because of the complexity of IMC and the importance of involving stakeholders in future solutions for providing health care, local competence and political management be encouraged to find shared values regarding the view of health care and common understanding of future projection and solutions to deal with IMC. Our study used the theory of coopetition to explain some of the challenges of IMC. The simultaneous appearance of cooperation and competition can be positive for municipalities, as well as in the private sector. More research is required to determine how this simultaneously affects IMC. People need to be aware of the presence of coopetition in IMC in health care services, so no inconsistencies appear in reforms and policies. Our study also clarifies the need for more research on IMC. In particular, our study indicates that tailored research on IMC is required. Research on the prevalence of challenges of IMC divided into different forms of organization will be an important follow-up and complementation of the present study.

## Competing interests

The authors declare they have no competing interests.

## Authors’ contributions

EHR carried out the Delphi-study, drafted the manuscript and performed statistical analysis and abstraction of the data. TE participated to the design of the study, interpretation of data and revising the manuscript. RF participated in the design of the study and revising the manuscript, ET participated in analysis of data and revising the manuscript. ÅS participated to the design of the study, interpretation of data and coordinated and revised the manuscript. All authors read and approved the final manuscript.

## Pre-publication history

The pre-publication history for this paper can be accessed here:

http://www.biomedcentral.com/1472-6963/13/451/prepub

## Supplementary Material

Additional file 1 Important challenges concerning coordination. Click here for file

Additional file 2 **Important challenges concerning inter-municipal collaboration.** Click here for file

Additional file 3 Statements presented in round four. Click here for file
