# Supplementary material for: Important challenges for coordination and inter-municipal cooperation in health care services: a Delphi study
Source: BMC Health Serv Res. 2013 Oct 30;13:451. doi: 10.1186/1472-6963-13-451 (PMC4228434; doi:10.1186/1472-6963-13-451)
Supplement: Additional file 1 — Important challenges concerning coordination. [file 1472-6963-13-451-S1.pdf]

Additional file 1: Important challenges concerning coordination

Category	Challenge	Identified as important by the experts N=11	Identified as important by more than half of the experts
**Education/ qualification**	Lack of competence on goal achievement.	4	
	Lack of/low coverage on required professional expertise/special skills.	3	
	Low workforce in municipal nursing and care sector.	4	
	Challenging to conduct skills development amongst employees to meet the new tasks.	1	
	Challenging to recruit and retain sufficient qualified personnel/ required skills.	4	
	One cannot meet new and more complicated patient/user situations.	1	
	Challenging to teach employees about cooperative agreements and the implications on their work.	5	
	Lack of knowledge in the organization concerning social planning as means of promoting public health.	2	
	Not enough expertise in the municipality concerning the coordination reform and new legislation.	1	
	Managers and staff lack collaborative skills.	3	
	Lack of interdisciplinary focus internally and externally on how to conduct the necessary competence enhancements.	4	

**Pressure on resources**	Services are under pressure due to limited resources.	4	
	Problems are transferred to other collaborating parts, one wishes that the "other" takes responsibility but does not understand that there are two equal partners.	4	
	The reform is not fully funded for the municipalities.	8	X
	Hard to invest and build new services.	4	
	Scarce resources in terms of time, personnel and funding in the municipality.	8	X
	Collaboration is not carried out in a good and effective manner.	0	
	Political decisions concerning downsizing in health and social sector in front of the coordination reform.	1	
	High level of employee turnover hinders the creation of good routines on evening, night and weekend shifts.	0	
**Collaboration**	All hospital employees do not relate to what has been decided related to collaboration with the municipality.	4	
	Internal collaboration problems at hospitals.	1	
	Changes and information comes faster than you are able to handle.	2	
	Lack of internal dialogue in the municipality.	0	
	Divergent understanding and view of	4	

	each other's tasks.		
	It is not taken into account that some municipalities are large enough to handle the tasks themselves.	2	
	Inter-municipal cooperation is perceived as imposed upon.	2	
	Challenging to get everyone in own municipality to pull in one direction.	6	X
	Challenging to obtain information flow.	7	X
**Cooperation with central government**	The government does not provide necessary and adequate information concerning tasks they impose on the municipality.	0	
	Challenging to perform tasks that authorities demand before a given deadline. Changes come faster than one can manage.	5	
	Challenging to ensure that collaborating parties have the same understanding in a dialogue.	5	
	Uncertainty regarding what to do yourself and what you should collaborate on.	4	
**Electronic information exchange**	Electronic messages are temporarily sent on epicrisis message format as a hybrid solution.	0	
	The hospitals have not prioritized the electronic messages supporting the process.	1	
	Lack of electronic communication.	10	X
	Lack of common tools for electronic communication, there is a problem with several suppliers.	6	X

	Electronic messages represent a fragile system.	2	
**Relationship between the municipality / specialist health care**	Uncertainty exists in the support to municipalities from mobile specialist health care teams.	3	
	The hospitals do not go into dialogue with the municipality.	2	
	Unclear division of tasks.	3	
	Specialist health care want to relate to fewest partners possible, and expect municipalities and regions to emerge as one partner towards the hospital.	4	
	Lack of synchronization concerning the patients we "think" will be a municipal responsibility.	5	
	Discharge of patients is a challenge.	2	
	Hospital staff may have poor knowledge of contracts/reforms.	7	X
	Patients being discharged are not prepared; information and documents relating to patients are missing when discharged from hospital.	5	
	Unrealistic dates are set for discharge of patient.	2	
	Hospital staff are difficult to contact by telephone.	3	
	The hospital sets the conditions for the process concerning discharge of patients.	6	x
	Patients are discharged earlier than before.	8	X
	Patients discharged are in worse	MISSED	

	health condition than they used to.		
	The hospital shows no respect for the competence in the municipality.	2	
	The specialist health service imposes changes that will directly affect the municipality, without involving the municipality in the process.	4	
	Challenging to achieve a common understanding of the purpose of the reforms.	4	
	Lack of understanding of each other's organizations.	5	
	The hospital does not ask what competence the municipalities possess.	4	
	Specialist health care still has the power to define many situations. Instead of collaboration, the municipality is ordered on what services the patient must have.	4	
	It is challenging to transfer knowledge from the specialist health care to the municipality.	2	
**Patient Focus**	Patient need is not well enough taken care of in specialist health care.	1	
	Decisions about patient services are influenced by economic considerations rather than concern for patients.	6	X
	Employees' need for interesting tasks and permanent employment are more important than the patients' needs.	1	
	Challenge to collaborate within the municipality with a focus on the best	1	

	interests of the patient.		
	Challenging to ensure that right patients are admitted to the regional medical centre.	1	
	Lack of resources for day care, activities, social meeting place for people living at home, especially lonely elderly people.	7	X
	Lack of focus on prevention.	7	X
	Challenging to get municipalities and hospitals to adopt a common focus on prevention rather than treatment.	4	
**Culture**	Challenging to create a cultural change in the municipality, so that one can facilitate interdisciplinary collaboration.	2	
	GPs and the municipality base their views on different information.	2	
	Specialist health service has focus on diagnosis and treatment, but the municipality has focused on coping and quality of life.	8	X
	Different cultures and different patient perspective in municipal and specialist health care services.	7	X
	Both politicians and the administration have a strong desire to "make do" in their own municipalities.	7	X
	Within the municipality there is a mismatch concerning what patient groups to prioritize.	1	
**Physical arrangements**	Challenging to get special assistive devices from technical aids centre in the municipality.	4	

	Lack of or too few special assistive devices in internal stock.	5	
	Challenging to obtain the necessary equipment and facilities to meet new patient-related tasks.	6	X
