# Supplementary material for: Important challenges for coordination and inter-municipal cooperation in health care services: a Delphi study
Source: BMC Health Serv Res. 2013 Oct 30;13:451. doi: 10.1186/1472-6963-13-451 (PMC4228434; doi:10.1186/1472-6963-13-451)
Supplement: Additional file 2 — Important challenges concerning inter-municipal collaboration. [file 1472-6963-13-451-S2.pdf]

Additional file 2: Important challenges concerning inter-municipal collaboration

Category	Challenge	Identified as important by the experts. N=9	Identified as important by more than half of the experts
**Localization / Distance**	Larger distances between services and inhabitants' homes and local support system than if the offer was made in their own municipality.	**7**	**X**
	Inconsistent use of the inter-municipal services.	**1**	
	It could become prestigious to localize the project in own district and closer to the clients. As a result, the choice of municipality to localize services is subject to political debate.	**6**	**X**
	Large geographic distances between municipalities.	**1**	
**Organizing**	The largest of the cooperating municipalities must lead many of the processes. As a result, small municipalities claim they lose control and influence. Maintaining the interest of small municipalities can be challenging.	**3**	
	Political leadership and management of inter-municipal work are demanding and require more than only organizing the municipal services.	**5**	**X**
	Administrative challenges for the participating municipalities related to the economy and decision making.	**7**	**X**
	Decisions on how to best organize inter-municipal work is challenging. There are different models with different advantages and	**6**	**X**

	disadvantages.		
	Clear responsibilities and lines of authority can be challenging to establish.	**4**	
	Municipalities are very different; it is often challenging to find common solutions.	**3**	
	Different organizing makes it hard to find the effective level of cooperation for the administration.	**2**	
**Documentation Systems**	Challenging when someone needs to replace the documentation system.	**3**	
	Various documentation systems that cannot be integrated.	**6**	**X**
	Different understanding and different tools.	**4**	
	Different basic data in IPLOS.	**2**	
**Economy**	Inter-municipal work tends to be expensive, as no one "owns" it.	**5**	**X**
	Challenging to agree on cost allocation, number of places and administration.	**6**	**X**
**Establishment of internal municipal services**	Challenging to find suitable areas for cooperation.	**3**	
	It can often be a long way from idea to decision.	**7**	**X**
	When considering inter-municipal work, most municipalities think "to the necessary extent" rather than to "the sufficient extent".	**4**	
	It is challenging to establish inter-municipal cooperation as it is often more tempting to solve problems	**9**	**X**

	alone since this is more flexible, it creates synergy, and expertise that can be applied across the municipality.		
	Municipalities are lacking skills, ability to recruit and offer fragmented services.	**2**	
**Interests / priorities**	Partly different interests.	**5**	**X**
**Highlight inter-municipal services**	Challenging to distribute knowledge in the organization, and to anchor the joint cooperation projects at the local level.	**7**	**X**
**Staffing / Expertise**	Challenges occur in the municipality when staffing levels decrease as a result of the work being resolved within the inter-municipal service.	**4**	
	Professional environment/ challenges are "removed" from some municipalities and centralized. It can lead to impoverishment in some municipalities.	**5**	**X**
