# Supplementary material for: Important challenges for coordination and inter-municipal cooperation in health care services: a Delphi study
Source: BMC Health Serv Res. 2013 Oct 30;13:451. doi: 10.1186/1472-6963-13-451 (PMC4228434; doi:10.1186/1472-6963-13-451)
Supplement: Additional file 3 — Statements presented in round four. [file 1472-6963-13-451-S3.pdf]

Additional file 3: Statements presented in round four

Q-1	Q-2
1. Challenging to obtain the necessary equipment and facilities to meet new patient-related tasks.	1. Larger distances between services and inhabitants' homes and local support system than if the offer was made in their own municipality.
2. Politicians have a strong desire to "make do" in their own municipalities.	2. It could become prestigious to localize the project in own district and closer to the clients. As a result, the choice of municipality to localize services is subject to political debate.
3. Different culture in municipal and specialist health care services.	3. Political leadership and management of inter-municipal work are demanding and require more than only organizing the municipal services.
4. Specialist health service has focus on diagnosis and treatment, but the municipality has focused on coping and quality of life.	4. Administrative challenge for the participating municipalities related to the economy and decision making.
5. Lack of focus on prevention.	5. Decisions on how to best organize inter-municipal work is challenging. There are different models with different advantages and disadvantages.
6. Lack of resources for day care (e.g. activities, social meeting place for people living at home, especially lonely elderly people).	6. Various documentation systems that cannot be integrated.
7. Decisions concerning patient services are	7. Inter-municipal work tends to be expensive, as

influenced by economic considerations rather than concern for patients.	no one "owns" it.
8. Hospital staff may have poor knowledge of contracts / reforms.	8. Challenging to agree on cost allocation.
9. Lack of electronic communication.	9. It can often be a long way from idea to decision.
10. Lack of common tools for electronic communication.	10. It is challenging to establish inter-municipal cooperation as it is often more tempting to solve problems alone since this is more flexible, it creates synergy, and expertise that can be applied across the municipality.
11. Patients are discharged earlier than before.	11. Partly different interests.
12. The hospital sets the conditions for the process concerning discharge of patients.	12. Challenging to anchor the joint cooperation projects at the local level.
13. The reform is not fully funded for the municipalities.	13. Professional environment/ challenges are "removed" from some municipalities and centralized. It can lead to impoverishment in some municipalities.
14. Scarce resources in terms of time.	14. Administrative challenge for the participating municipalities related to the decision making.
15. Challenging to get everyone in own municipality to pull in one direction.	15. Challenging to distribute knowledge about joint cooperation projects in the organization.
16. Challenging to obtain information flow.	16. Challenging to agree on number of places.
17. Different patient perspective in municipal and specialist health care services.	17. Challenging to agree on administration.

18. Scarce resources in terms of personnel.	
19. Scarce resources in terms of funding.	
20. The administration has a strong desire to "make do" in their own municipality	
21. Patients discharged are in worse health condition than they used to.<br>(Was only to be rated, if they would have chosen it in round three)	
